# Identifying Facemask-Wearing Condition Using Image Super-Resolution with Classification Network to Prevent COVID-19

**DOI:** 10.3390/s20185236

**Published:** 2020-09-14

**Authors:** Bosheng Qin, Dongxiao Li

**Affiliations:** College of Information Science and Electronic Engineering, Zhejiang University, Hangzhou 310058, China; 3170105600@zju.edu.cn

**Keywords:** facial recognition, convolutional neural network, image super-resolution, facemask-wearing condition, deep learning, SRCNet, COVID-19

## Abstract

The rapid worldwide spread of Coronavirus Disease 2019 (COVID-19) has resulted in a global pandemic. Correct facemask wearing is valuable for infectious disease control, but the effectiveness of facemasks has been diminished, mostly due to improper wearing. However, there have not been any published reports on the automatic identification of facemask-wearing conditions. In this study, we develop a new facemask-wearing condition identification method by combining image super-resolution and classification networks (SRCNet), which quantifies a three-category classification problem based on unconstrained 2D facial images. The proposed algorithm contains four main steps: Image pre-processing, facial detection and cropping, image super-resolution, and facemask-wearing condition identification. Our method was trained and evaluated on the public dataset Medical Masks Dataset containing 3835 images with 671 images of no facemask-wearing, 134 images of incorrect facemask-wearing, and 3030 images of correct facemask-wearing. Finally, the proposed SRCNet achieved 98.70% accuracy and outperformed traditional end-to-end image classification methods using deep learning without image super-resolution by over 1.5% in kappa. Our findings indicate that the proposed SRCNet can achieve high-accuracy identification of facemask-wearing conditions, thus having potential applications in epidemic prevention involving COVID-19.

## 1. Introduction

Coronavirus disease 2019 (COVID-19) is an emerging respiratory infectious disease caused by Severe Acute Respiratory Syndrome coronavirus 2 (SARS-CoV2) [1]. At present, COVID-19 has quickly spread to the majority of countries worldwide, affecting more than 14.9 million individuals, and has caused 618,017 deaths, according to the report from the World Health Organization (WHO) on 23 July 2020 (https://covid19.who.int/). To avoid global tragedy, a practical and straightforward approach to preventing the spread of the virus is urgently desired worldwide.

Previous studies have found that facemask-wearing is valuable in preventing the spread of respiratory viruses [2,3,4]. For instance, the efficiencies of N95 and surgical masks in blocking the transmission of SARS are 91% and 68%, respectively [5]. Facemask-wearing can interrupt airborne viruses and particles effectively, such that these pathogens cannot enter the respiratory system of another person [6]. As a non-pharmaceutical intervention, facemask-wearing is a non-invasive and cheap method to reduce mortality and morbidity from respiratory infections. Since the outbreak of COVID-19, facemasks have been routinely used by the general public to reduce exposure to airborne pathogens in many countries [7]. In addition to patients suspected of actual infection with COVID-19 being required to wear facemasks for the prevention of virus spreading, healthy persons also need to wear facemasks in order to protect themselves from infection [1]. Facemasks, when fitted properly, effectively disrupt the forward momentum of particles expelled from a cough or sneeze, preventing disease transmission [5]. However, the effectiveness of facemasks in containing the spread of airborne diseases in the general public has been diminished, mostly due to improper wearing [8]. Therefore, it is necessary to develop an automatic detection approach for facemask-wearing condition, which can contribute to personal protection and public epidemic prevention.

The distinctive facial characteristics in facemask-wearing conditions provide an opportunity for automatic identification. Recent rapid technological innovations in deep learning and computer vision have presented opportunities for development in many fields [9,10]. As the main component of deep learning methods, deep neural networks (DNNs) have demonstrated superior performance in many fields, including object detection, image classification, image segmentation, and distancing detection [11,12,13,14,15,16]. One primary model of DNNs is convolutional neural networks (CNNs), which have been widely used in the field of computer vision tasks. After training, CNNs can recognize and classify facial images—even with slight differences—due to their powerful feature extraction capability. As one of the CNNs, image super-resolution (SR) networks can restore image details. Recently, SR networks have become more in-depth, and the ideas of auto-encoder and residual learning have been integrated for performance improvement [17,18]. SR networks have also been applied for image processing before image segmentation or classification, reconstructing images for higher resolution and restoring details [19,20,21,22,23]. Moreover, SR networks can improve the classification accuracy significantly, especially when using a dataset with low-quality images, and provide a feasible solution to improve facemask-wearing condition identification performance. Therefore, the combination of an SR network with a classification network (SRCNet) could be utilized in facial image classification for accuracy improvement.

To our knowledge, there have not been any published reports related to SR networks combined with classification networks for accuracy improvement in facial image classification, especially regarding the automatic detection of facemask-wearing conditions. Therefore, we intend to develop a novel method combining an SR network with a classification network (SRCNet) to identify facemask-wearing conditions, in order to improve classification accuracy with low-quality facial images.

Our main contributions can be summarized as follows.

(1) Development of a new face accessory identification method that combines an SR network with a classification network (SRCNet) for facial image classification.
(2) Utilization of a deep learning method for automatic identification of facemask-wearing conditions. To our knowledge, this is the first time a deep learning method has been applied to identifying facemask-wearing condition.
(3) Improving the SR network structure, including activation functions and the density of skip connections, which outperforms previous state-of-the-art methods.

## 2. Related Work

### 2.1. Image Super-Resolution

The idea of reconstructing high-quality images from low-resolution images has a long history. Bicubic was one of the most widely used methods, which up-sampled low-resolution images by linear interpolation in both the *x*-axis and *y*-axis. However, the reconstructed images using the bicubic method were blurred, due to the loss of high-frequency information. Hence, high-performance algorithms have been introduced. Yang, et al. [24] presented an SR method based on sparse representation, which used sparse representations for each patch of the low-resolution input and then calculated the coefficients to generate a high-resolution output. The example-based SR method was introduced by Timofte, et al. [25], which reconstructs images based on a dictionary of low-resolution and high-resolution exemplars. Recently, deep learning methods have also been introduced for SR [26,27,28,29,30,31,32,33,34]. Dong, et al. [35] first presented the SRCNN, which utilized a three-layer CNN for image super-resolution, after which more high-performance network structures have been introduced for SR, such as VDSR [29] and RED [17]; VDSR increases the depth of the CNN in SR and proposes residual learning for fast training, while RED introduces symmetric convolutional layers and deconvolutional layers with skip connections for better performance.

### 2.2. Classification Network

Deep learning methods have outperformed traditional image classification networks in many aspects, especially using the CNN algorithm. Tuning CNNs for better accuracy has been an area of intensive research over the past several years, and some high-performance CNN architectures (e.g., AlexNet [13], VGGNet [36], GoogLeNet [37], and ResNet [38]) have been introduced. Recently, the tuning of a CNN has progressed in two separate ways: One drawing representational power from deeper or wider architectures by increasing the number of trainable parameters (e.g., Inception-v4 [39], Xception [40], and DenseNet [41]), while other research has focused on building small and efficient CNNs due to limitations in computational power (e.g., MobileNet [42], MobileNet-v2 [43], ShuffleNet [44], and SqueezeNet [45]). All of these network structures outperformed the traditional machine learning methods, such as Histograms of Oriented Gradient (HOG)-based support vector machines (SVM) [46] and K-nearest Neighbors (kNN), in classification tasks using either the ImageNet classification dataset [47] or the CIFAR-10 classification dataset [48].

As CNNs have become deeper and wider, overfitting problems have been raised, mainly due to the limitations of datasets, which are detrimental to the generalization of networks. To prevent overfitting, one way is to change the architecture of neural networks, for example, by adding dropout layers [49]. Some studies have focused on the hyper-parameters in training options and adding regularization terms [38,50,51]. Data augmentations such as random rotation, random cropping, and random reflections have also been widely applied for prevention of the overfitting problem [13,38,41,42,52].

### 2.3. Image Super-Resolution for Classification

Upscaling low-quality (low-resolution, blurred) input images to produce high-quality feature maps to improve classification performance is one of the most popular ways for low-quality image classification or object detection [53,54,55,56,57]. Na, et al. [56] introduced an SR method on cropped regions or candidates to improve object detection and classification performances. Cai, et al. [57] has developed a resolution-aware convolutional deep model combining super-resolution and classification. SR was also applied in facial recognition. Zou, et al. [58] adopted SR to improve facial recognition performance on low-resolution images, proving that the combination of SR and a facial recognition model concurrently allows for increased recognition performance. Uiboupin, et al. [59] adopted SR using sparse representation for improving face recognition in surveillance monitoring. However, these SR methods for improving face recognition accuracy are either based on facial features or high-level representations. There have not been any published reports related to deep-learning-based SR networks combined with classification networks for accuracy improvement in facial image classification, especially regarding the automatic detection of facemask-wearing conditions. Hence, the SRCNet, combining a SR network and classification network, is proposed and utilized in facial recognition.

### 2.4. Face Accessories Detection

There is plenty of research using image features, machine learning, or deep learning methods for face accessories detection, especially in the area of glasses and hat detection. Jing, et al. [60] used image edge information in a small area between the eyes for glasses detection. Machine learning methods like SVM and kNN were also widely applied in face accessories detection [61,62,63]. Recently, the deep learning methods have become more prevalent in face accessories detection, where high-level and abstract information could be extracted through CNNs [64,65]. However, as one of the most common face accessories, there is a paucity in automatic facemask-wearing condition identification, especially using the deep learning method. Hence, the SRCNet is proposed to identify facemask-wearing condition, which has the application value, especially in epidemic prevention involving COVID-19.

## 3. Materials and Methods

This section describes the technology behind the SRCNet and facemask-wearing condition identification, including the proposed algorithm, image pre-processing, facial detection and cropping, SR network, facemask-wearing condition identification network, datasets, and training details. Facemask-wearing condition identification is a kind of three-category classification problem, including no facemask-wearing (NFW), incorrect facemask-wearing (IFW), and correct facemask-wearing (CFW). Our goal is to form a facemask-wearing condition identification function, FWI(x), which inputs an unprocessed image and outputs the conditions of wearing facemasks for all faces in the image.

### 3.1. Proposed Algorithm

**Figure 1:**
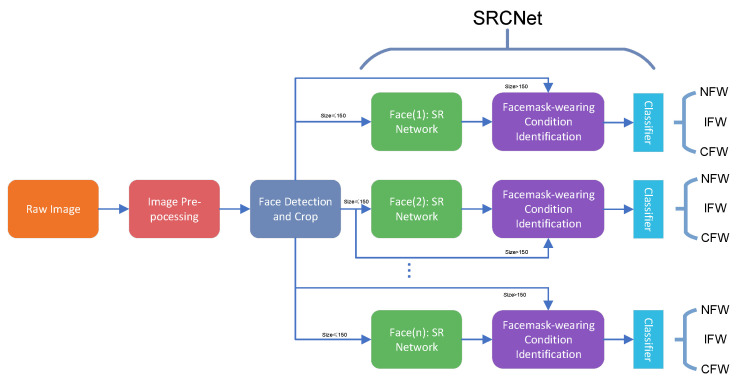
Diagram of the proposed algorithm. Face (n) is the $n^{th}$ cropped facial image. Listed super-resolution (SR) networks and facemask-wearing identification networks are to show that the SR and facemask-wearing condition identification process can both be executed in parallel for a single image with many faces. However, only one SR network and one facemask-wearing identification network are used in the proposed method. NFW = no facemask-wearing, IFW = incorrect facemask-wearing, CFW = correct facemask-wearing.

Figure 1 offers the diagram of the proposed algorithm, which contains three main steps: Image pre-processing, facial detection and cropping, and SRCNet for SR and facemask-wearing condition identification. After the pre-processing of raw images, all facial areas of images are detected using a multitask cascaded convolutional neural network [12]. The facial areas are then cropped, where the sizes of the cropped images vary. All cropped images are then sent to SRCNet for facemask-wearing condition identification. In SRCNet, all images are judged for the need of SR. As the size of the input images for the facemask-wearing condition identification network is 224 × 224, cropped images with a size no larger than 150 × 150 (i.e., width or length no more than 150) are sent to the SR network, and then for facemask-wearing condition identification. Otherwise, the cropped images are then directly sent for facemask-wearing condition identification. The output is the probabilities of the input images with respect to the three categories: NFW, IFW, and CFW. After passing through the classifier, the pipeline outputs the final facemask-wearing condition results.

### 3.2. Image Pre-Processing

**Figure 2:**
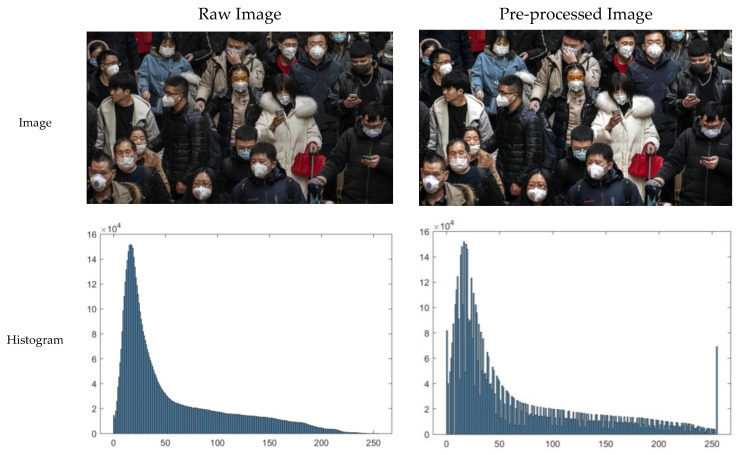
Diagram image pre-processing and corresponding histogram. The illumination and contrast of the image are higher through the image pre-processing process, where more details are visible. The original image was from the Medical Masks Dataset (https://www.kaggle.com/vtech6/medical-masks-dataset).

The goal of image pre-processing is to improve the accuracy of the following facial detection and facemask-wearing condition identification steps. SRCNet is designed to be applied in public for classification, taking uncontrolled 2D images as input. The raw images taken in real-life have considerable variance in contrast and exposure, so image pre-processing is needed to ensure the accuracy of facial detection and facemask-wearing condition identification [66]. From our experiment, the face detector is likely to make errors when images are underexposed. The raw images were adjusted, using the MATLAB image processing toolbox, by mapping the values of the input intensity image to the new value, in which 1% of the values are saturated at low and high intensities of the input data. The image pre-processing diagram and corresponding histogram are illustrated in Figure 2.

### 3.3. Facial Detection and Cropping

As SRCNet needs to concentrate on the information from faces, rather than the background, in order to improve accuracy, a face detector is needed for the detection of faces and to crop facial areas. The uncontrolled 2D images have differences in face size, expression, and background. Hence, a robust and highly accurate face detector is needed. The multitask cascaded convolutional neural network was adopted for facial detection, which has been shown to perform well in obtaining facial areas in real environments [12].

**Figure 3:**
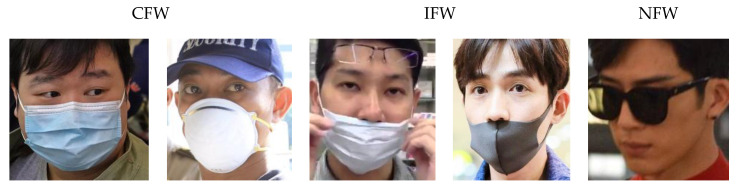
Examples of cropped facial images. The bounding boxes are set with some background left to avoid the face detector making bias. From our experiment, the bounding boxes are likely to have a slight drift when people are not directly facing the camera. Larger bounding boxes ensure that the facial areas are correctly cropped. Besides, more facial information is preserved with large bounding boxes. The original image was from the Medical Masks Dataset (https://www.kaggle.com/vtech6/medical-masks-dataset).

After obtaining the position of the face, faces are then cropped from the pre-processed image, to serve as the inputs of the SR network or facemask-wearing condition identification network, depending on image sizes. Image sizes no more than 150 × 150 (width or length no more than 150) were first input to the SR network, and then for facemask-wearing condition identification. Other cropped facial images were directly sent to the facemask-wearing condition identification network. Examples of cropped images are shown in Figure 3.

### 3.4. SR Network

**Figure 4:**
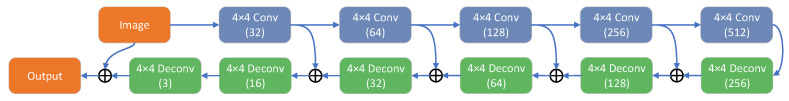
Structure of SR network.

The first stage of SRCNet is the SR network. The cropped facial images have a huge variance in size, which could possibly damage the final identification accuracy of SRCNet. Hence, SR is applied before classification. The structure of the SR network was inspired by RED [17], which uses convolutional layers as an auto-encoder and deconvolutional layers for image up-sampling. Symmetric skip connections were also applied to preserve image details. The detailed architectural information of the SR network is shown in Figure 4.

The SR network has five convolutional layers and six deconvolutional layers. Except for the final deconvolutional layer, all other convolutional layers are connected to their corresponding convolutional layers by skip connections. With skip connections, the information is propagated from convolutional feature maps to the corresponding deconvolutional layers and from input to output. The network is then fitted by solving the residual of the problem, which is denoted as

(1) $$F_{i}\left(X\right)=GT_{i}-I_{i}$$

where $GT_{i}$ is the ground truth, $I_{i}$ is the input image, and $F_{i}\left(X\right)$ is the function of the SR network for the $i^{th}$ image.

In convolutional layers, the number of kernels was designed to increase by a factor of 2. With kernels size 4 × 4 and stride 2, after passing through the first convolutional layer for feature extraction, every time the image passes through a convolutional layer, the size of the feature maps decreases by a factor of ½. Hence, the convolutional layers act as an auto-encoder and extract features from the input image.

In the deconvolutional layers, the number of output feature maps is symmetric to the corresponding convolutional layers, in order to satisfy the skip connections. The number of kernels in every deconvolutional layer decreases by a factor of ½ (except for the final deconvolutional layer), while, with kernels size 4 × 4 and stride 2, the size of feature maps increases by a factor of 2. After information combination in the final deconvolutional layer, the output is an image with the same size as the input image. The deconvolutional layers act as a decoder, which take the output of the encoder as input and up-sample them to obtain a super-resolution image.

It is worth mentioning that the function used for down-and up-sampling is the stride in the convolutional and deconvolutional layers—rather than pooling and un-pooling layers—as the aim of the SR network is to restore image details rather than learning abstractions (pooling and un-pooling layers damage the details of images and deteriorate the restoration performance [17].

The function of the final deconvolutional layer is to combine all the information from the previous deconvolutional layer and input image and normalize all pixels to [0, 1] as the output. The stride for the final deconvolutional layer was set to 1, for information combination without up-sampling. The activation function of the final deconvolutional layer is Clipped Rectified Linear Unit, which forces normalization of the output and avoids error in computing the loss. The definition of Clipped Rectified Linear Unit is as follows:

(2) ClippedReLU(x)=min(1,max(0,x))

where *x* is the input value.

One main difference between our model and RED is the improvement in the activation functions, which was changed from a Rectified Linear Unit (ReLU) to a Leaky Rectified Linear Unit (LeakyReLU) for all convolutional and deconvolutional layers except the final deconvolutional layer, which use Clipped Rectified Linear Unit as the activation function to limit values in the range [0, 1]. Previous studies have shown that different activation functions have an impact on the final performance of a CNN [67,68]. Hence, the improvement in the activation functions contributed to the better image restoration by the SR network. The ReLU and LeakyReLU are defined as follows:

(3) ReLU(x)=max(0,x)

(4) LeakyReLU(x)=max(0,x)+min(0,α×x)

where *x* is the input value and α is a scale factor.

The reason for this improvement was that the skip connections propagated the image from input to output. For an SR network, the network shall have the capability to subtract or add values for pixels, where the ReLU function can only add values for feature maps. The LeakyReLU function, however, can activate neurons with negative values, thus improving the performance of the network.

Another difference is the density of skip connections. Rather than using skip connections every few (e.g., two in RED) layers from convolutional layers to their symmetrical deconvolutional feature maps, the density of skip connections increased, and all convolutional layers were connected to their mirrored deconvolutional layers. The reason for this was to cause all layers to learn to solve the residual problem, which reduced the loss of information between layers while not significantly increasing the network parameters.

The goal of SR network training is to update all learnable parameters to minimize the loss. For SR networks, the mean squared error (MSE) is widely used as the loss function [17,27,34,35]. A regularization term (weight decay) for the weights is added to the MSE loss to reduce overfitting. The MES with $L_{2}$ regularization was applied as the loss function Loss(w), which is defined as

(5) $$Loss\left(w\right)=\frac{1}{N}\overset{N}{\underset{i\;=\;1}{\sum }}{\left|\left|GT_{i}-O_{i}\right|\right|}^{2}{}_{F}+\frac{1}{2}\times \lambda \times w^{T}w$$

where $GT_{i}$ is the ground truth, $O_{i}$ is the output image, and Loss(w) is the loss for collections of given w.

It is worth mentioning that the size of the input image can be arbitrary and that the output image has the same size as the input image. The convolutional and deconvolutional layers are symmetric for the SR network. Furthermore, the network is predicted pixel-wise. For better detail enhancement, we chose a dedicated image input size for SR network training and, so, the input images were resized to 224 × 224 × 3 with bicubic interpolation (which was the same as the input image size of the facemask-wearing condition identification network). The output of the SR network is enhanced images with the same size of the inputs (224 × 224 × 3), and the enhanced images will be sent directly to the facemask-wearing condition identification network for classification.

### 3.5. Facemask-Wearing Condition Identification Network

The second stage of SRCNet is facemask-wearing condition identification. As CNNs are one of the most common types of network for image classification, which perform well in facial recognition, a CNN was adopted for the facemask-wearing condition identification network in the second stage of SRCNet. The goal was to form a function G(FI), where FI is the input face image, which outputs the probabilities of the three categories (i.e., NFW, IFW, and CFW). The classifier then outputs the classification result based on the output possibilities.

MobileNet-v2 was applied as the facemask-wearing condition identification network, which is a lightweight CNN that can achieve high accuracy in image classification. The main features of MobileNet-v2 are residual blocks and depthwise separable convolution [42,43]. The residual blocks contribute to the training of the deep network, addressing the gradient vanishing problem and achieving benefits by back-propagating the gradient to the bottom layers. As for facemask-wearing condition identification, there are slight differences between IFW and CFW. Hence, the capability of feature extraction or the depth of the network are essential, contributing to the final identification accuracy. Depthwise separable convolution is applied for the reduction of computation and model size while maintaining the final classification accuracy, which separable convolution splits into two layers: One layer for filtering and another layer for combining.

Transfer learning is applied in the network training procedure, which is a kind of knowledge migration between the source and target domains. The network is trained in three steps: Initialization, forming a general facial recognition model, and knowledge transfer to facemask-wearing condition identification. The first step is initialization, which contributes to the final identification accuracy and training speed [38,69]. Then, a general facial recognition model is formed using a large facial image dataset, where the network gains the capability of facial feature extraction. After watching millions of faces, the network then concentrates on facial information, rather than the interference from backgrounds and the differences caused by image shooting parameters. The final step is knowledge transfer between facial recognition and facemask-wearing condition identification. The final fully connected layer is modified to meet with category requirements of facemask-wearing condition identification.

The reason for adopting transfer learning was the considerable differences in data volumes and their consequences. The facemask-wearing condition identification dataset is relatively small, compared to general facial recognition datasets, which may cause overfitting problems and a reduction in identification accuracy during the training process. Hence, the network gains knowledge about faces in the general facial recognition model training process for the reduction in overfitting and the improvement in accuracy.

The final stage of the classifier is the softmax function, which calculates the probabilities of all classes using the outputs of its direct ancestor (i.e., fully connected layer neurons) [70]. The definition is:

(6) $$p_{i}=\frac{e^{x_{i}}}{\sum_{j}e^{x_{j}}}$$

where $x_{i}$ is the total input received by unit i and $p_{i}$ is the prediction probability of the image belonging to class i.

The training goal of the facemask-wearing condition identification network was to minimize the cross-entropy loss with weight decay. For image classification, cross-entropy is widely used as the loss function [71,72]. A regularization term (weight decay) can help to significantly avoid overfitting. Hence, cross-entropy with $L_{2}$ regularization was applied as the loss function $Loss_{R}$, defined as

(7) $$Loss_{R}=-\overset{N}{\underset{i\;=\;1}{\sum }}\overset{K}{\underset{j\;=\;1}{\sum }}t_{ij}\log\left(y_{ij}\right)+\frac{1}{2}\times \lambda \times w^{T}w$$

For the cross-entropy term, N is the number of samples, K is the number of classes, $t_{ij}$ is the indicator that the $i^{th}$ sample belongs to the $j^{th}$ class (which is 1 when labels correspond and 0 when they are different), and $y_{ij}$ is the output for sample i for class j, which is the output value of the softmax layer. For the cross-entropy term, w is the learned parameters in every learned layer and λ is the regularization factor (coefficient).

### 3.6. Datasets

Different facial image data sets were used for different network training for the improvement in the generalization ability of SRCNet. The public facial image dataset CelebA was processed and used for SR network training [73]. As the goal of the SR network was detail enhancement, a large and high-resolution facial image data set was needed; CelebA met these requirements.

The processing of CelebA included three steps: Image pre-processing, facial detection and cropping, and image selection. All raw images were pre-processed as mentioned above. The facial areas were then detected by the multitask cascaded convolutional neural network and cropped for training, as the SR network was designed for restoring detailed information of faces rather than the background. Cropped images that were smaller than 224 × 224 (i.e., the input size of the facemask-wearing condition identification network) or non-RGB images were discarded automatically. All other cropped facial images were inspected manually and images with blur or dense noise were also discarded. Finally, 70,534 high-resolution facial images were split into a training dataset (90%) and a testing dataset (10%) and were adopted for SR network training and testing.

Training of the facemask-wearing condition identification network comprised three steps. Each step used a different data set for training. For initialization, the goal was generalization. A large-scale classification data set was needed for better generalization and, so, the ImageNet dataset was adopted for network initialization [13]. During this procedure, non-zero values were assigned to parameters, which increased the generalization ability. Furthermore, proper initialization significantly improves the training speed and better informs the general facial recognition model.

The general facial recognition model was trained with a large-scale facial recognition database, the CASIA WebFace facial dataset [74]. All images were screened manually and those containing insufficient subjects or with poor image quality were discarded [74]. Finally, the large-scale facial recognition dataset contained 493,750 images with 10,562 subjects, which was split into a training data set (90%) and testing data set (10%). The training set was applied for general facial recognition model training.

The public facemask-wearing condition dataset Medical Masks Dataset (https://www.kaggle.com/vtech6/medical-masks-dataset) was applied for fine-tuning the network, in order to transfer knowledge from general facial recognition to facemask-wearing condition identification. The 2D RGB images were taken in uncontrolled environments, and all faces in the data set had their position co-ordinates with facemask-wearing condition labels. The Medical Masks Data set was processed in four steps: Facial cropping and labeling, label confirmation, image pre-processing, and SR. All faces were cropped and labeled using the given position coordinates and labels. All cropped facial images were then screened manually and those with incorrect labels were discarded. Then, the facial images were confirmed and pre-processed using the methods mentioned in Section 3.2. For the final accuracy of SRCNet, the data set was expanded for the case of not wearing a mask. The resolution of pre-processed images varied, as shown in Table 1. For accuracy improvement of the facemask-wearing condition identification network, the facial image must contain enough details. Hence, the SR network was applied to add details to low-quality images. Images of sizes no larger than 150 × 150 (i.e., width or length no more than 150) were processed using the SR network. Finally, the dataset contained 671 images of NFW, 134 images of IFW, and 3030 images of CFW. The whole dataset was separated into a training dataset (80%) and a testing dataset (20%) for facemask-wearing condition identification network training and testing.

### 3.7. Training Details

The training of SRCNet contained two main steps: SR network training and facemask-wearing condition identification network training.

For SR network training, the training goal was to restore facial details, which we used the training set of CelebA to achieve. Based on the characteristics of the Medical Masks Dataset, the input images were pre-processed to imitate the low-quality images in the Medical Masks Dataset. The high-resolution processed images in CelebA were first filtered with a Gaussian filter with a kernel size of 5 × 5 and a standard deviation of 10. Then, they were down-sampled to 112 × 112. As the size of the input and output was the same, the down-sampled images were then up-sampled to 224 × 224 with bicubic as input, with the same size as the input of the facemask-wearing condition identification network. Adam was adopted as the optimizer, with $\beta_{1}=0.9$, $\beta_{2}=0.999$, and $\epsilon =10^{-8}$ [75]. The network was trained for 200 epochs with an initial learning rate of $10^{-4}$ and with a learning rate dropping factor of 0.9 every 20 epochs. The mini-batch size was 48.

The first step of facemask-wearing condition identification network training was initialization. The network was trained using the ImageNet dataset, with the training parameters proposed in [43].

The second step was to form a general facial recognition model. The output classes were modified to match with the class numbers (10,562). For initialization, the weight and bias in the final modified fully connected layer were initialized using a normal distribution with 0 mean and 0.01 standard deviation. The network was trained for 50 epochs, with the training data set shuffled in every epoch. To increase the training speed, the learning rate drop was 0.9 for every 6 epochs with an initial learning rate of $10^{-4}$, which eliminated the problem of the loss becoming stable. The network was trained using Adam as the optimizer, with $\beta_{1}=0.9$, $\beta_{2}=0.999$, $\epsilon =10^{-8}$, and $10^{-4}$ weight decay for $L_{2}$ regularization, in order to avoid overfitting [75].

Transfer learning was applied for fine-tuning the facemask-wearing condition identification network, where the final fully connected layer and classifier were modified to match the classes (NFW, IFW, and CFW). The weights and biases in the final modified layer were initialized by independently sampling from a normal distribution with zero mean and 0.01 standard deviation, which produced superior results, compared to other initializers. Adam was chosen as the optimizer, while the learning rate was set as $10^{-4}$. To avoid overfitting, a $10^{-4}$ weight decay for $L_{2}$ regularization was also applied [75]. The batch size was set to 16 and the network was trained for 8 epochs in total. The grid search method was applied to search for the best combination of all the parameters mentioned above, in order to improve the performance of the facemask-wearing condition identification network.

Data augmentation can reduce the overfitting problem and contribute to the final accuracy of the network [36,52,76]. To train the general facial recognition network, the training dataset was randomly rotated in a range of 10° (in a normal distribution), shifted vertically and horizontally in a range of 8 pixels, and horizontally flipped in every epoch. During the fine-tuning stage, the augmentation was mild, with rotation within 6° (in normal distribution), shifting by up to 4 pixels (vertically and horizontally), and with a random horizontal flip in every epoch.

## 4. Results

SRCNet was implemented by MATLAB with the deep learning and image processing toolboxes for network training and image processing. A single Nvidia graphics processing unit (GPU) with the Nvidia CUDA deep neural network library (cuDNN) and compute unified device architecture (CUDA) was applied to implement SRCNet.

### 4.1. SR Network Experiment Results

For SR networks, the most widely used full-reference quality metrics are peak signal-to-noise ratio (PSNR) and structural similarity index (SSIM) [77]. The PSNR was used as the metric for quantitatively evaluating image restoration quality, while SSIM compared local patterns of pixel intensities for luminance and contrast.

Comparisons with previous state-of-the-art methods, including RED [17], SRCNN [35], VDSR [29], Lanczos [78], and bicubic, were made to illustrate the performance of the proposed SR network. All the methods were trained on the training set of CelebA (if needed) and tested on the testing set.

As in real applications, the quality of images varied. Low-quality images were mainly manifested in resolution and blur. Hence, different-quality images were simulated and used for testing the performance of the SR network, as carried out by changing the standard deviation σ, the size of Gaussian filters, and the resolutions. For testing with different standard deviations of Gaussian filters, the testing set was first filtered with Gaussian filters with a kernel size of 5 × 5 and standard deviations of 5, 10, 15, and 20, and then down-sampled to 112 × 112 for evaluation. For testing with different kernel sizes of Gaussian filters, the testing set was first filtered with Gaussian filters with kernel sizes of 3 × 3, 5 × 5, 7 × 7, and 9 × 9, and a standard deviation of 10, then down-sampled to 112 × 112 for evaluation. For testing with different image resolutions, the testing set was first filtered with a Gaussian filter with a kernel size of 5 × 5 and a standard deviation of 10, then down-sampled to 64 × 64, 96 × 96, 112 × 112, and 150 × 150 for evaluation.

The sizes of input images were the same as the outputs of the SR network. For evaluation of the effect of the SR network on the facemask-wearing condition identification network, which takes 224 × 224 images as input, all down-sampled testing sets were up-sampled to 224 × 224 as the input of the SR network. The evaluation results are shown in Table 2, Table 3 and Table 4. Compared to previous state-of-the-art methods, the proposed SR network performed better, especially in terms of SSIM.

As it can be observed from Table 4, after the size of the image reached 150 × 150, the performance of the network decreased. The reason for this was that the network was trained to restore blurred images with low resolution. With the increase in image resolution, the resolution and detail of facial images increased, which undermined the condition of using the network. Hence, only images with a size no larger than 150 × 150 (width or length no more than 150) were processed with the SR network. In this case, the SR network significantly outperformed bicubic.

**Figure 5:**
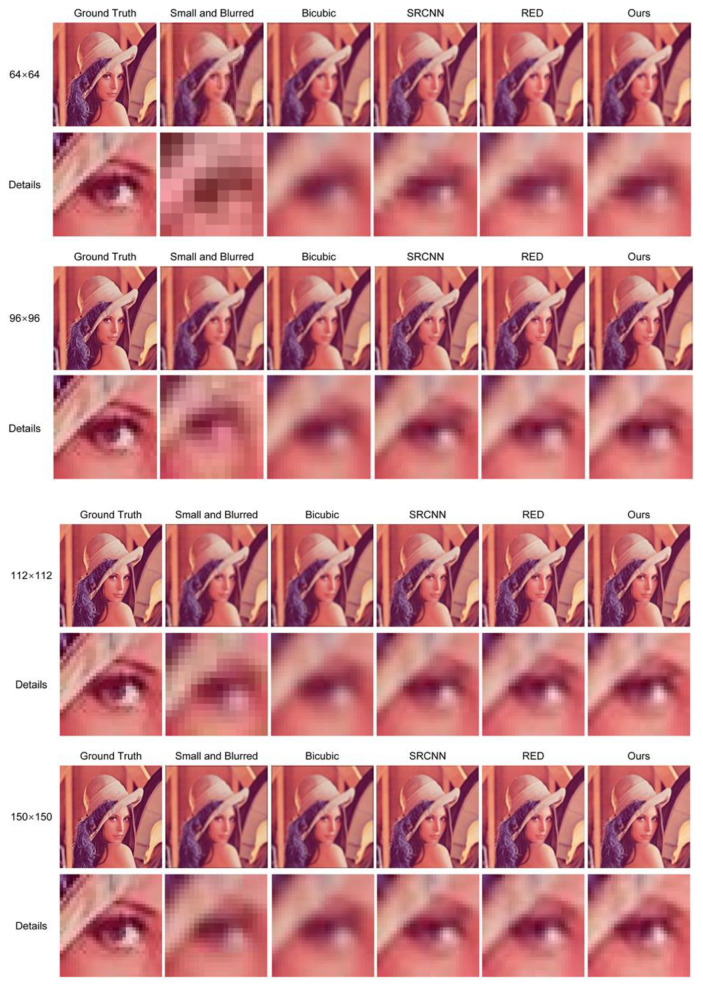
Visualization result with 64 × 64, 96 × 96, 112 × 112, and 150 × 150 blurred images. The details of images were highlighted.

As the images in the Medical Masks Dataset have a considerable variance in resolution, the SR network had to have good performance under different resolutions. Hence, different SR methods were compared and visualized with different resolutions of small and blurred images [79]. The testing image was first blurred with a Gaussian filter with a kernel size of 5 × 5 and a standard deviation of 10, then down-sampled to 64 × 64, 96 × 96, 112 × 112, and 150 × 150, respectively, before restoration. The visualized results are shown in Figure 5. Although all SR methods enhanced facial details, the proposed SR network outperformed other methods in all resolutions. The images restored by the proposed SR network were closer to the ground truth, due to its high PSNR and SSIM values.

### 4.2. Facemask-Wearing Condition Identification Network Comparison

To illustrate the advantages and reason for using MobileNet-v2 as the facemask-wearing condition identification network, comparisons with other CNNs, including Inception-v3 [80], DenseNet201 [41], ResNet50 [38], DarkNet19 [81], Xception [40], and VGG19 [36], in terms of network parameters and running time for a single image, were conducted, as shown in Table 5. Generally, the performance of a network increases with the depth of the network. MobileNet-v2 showed great performance for real-time identification, with low storage space and running time. In addition, the depth of MobileNet-v2 was deep, which contributed to its final performance in identifying facemask-wearing condition. From our experiment, MobileNet-v2 did not show a performance decrease compared to other networks, with the final facemask-wearing condition identification accuracy gap being less than 1%, compared to the other networks with the SR network. All experiments were conducted with MATLAB 2020a, a i7 CPU, and P600 GPU with 4 GB memory.

### 4.3. SRCNet Results

After training, SRCNet was tested using the testing set of the Medical Masks Dataset. The proposed algorithm was tested using an ablation experiment. The comparison in accuracy and the confusion matrix of SRCNet are reported.

The ablation experiment was designed to illustrate the importance of transfer learning and the SR network. The performances of SRCNet with or without transfer learning or the proposed SR network were compared, as shown in Table 6. Transfer learning and the SR network increased the identification accuracy considerably, by reducing the overfitting problem and increasing facial details, respectively. Finally, SRCNet reached an accuracy of 98.70% and outperformed MobileNet-v2 without transfer learning or the SR network by over 1.5% in kappa.

**Figure 6:**
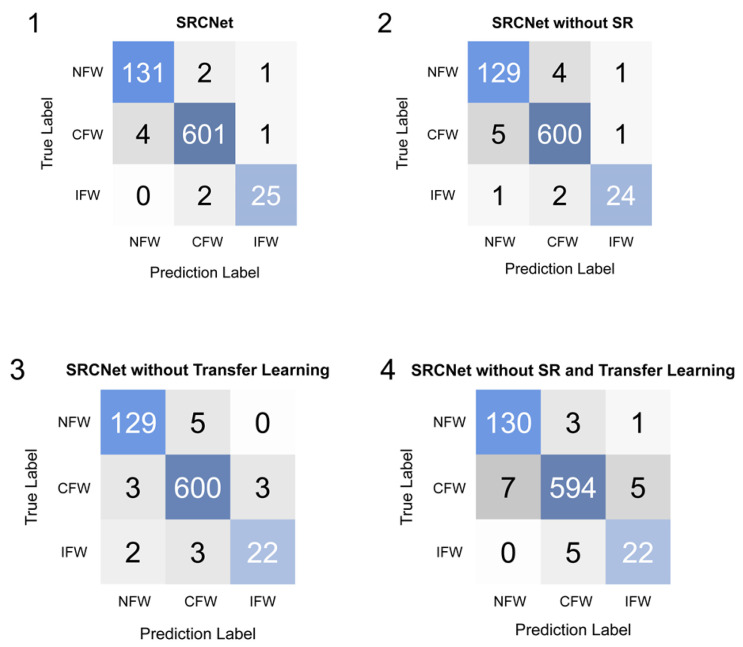
Comparison in confusion matrix. NFW = no facemask-wearing, IFW = incorrect facemask-wearing, CFW = correct facemask-wearing. **1**: Proposed SRCNet. **2**: Proposed SRCNet without SR network, which was an end-to-end facemask-wearing condition identification network with transfer learning. **3**: Proposed SRCNet without transfer learning. **4**: Proposed SRCNet without transfer learning or SR network, which was an end-to-end facemask-wearing condition identification network without transfer learning. All other settings, including hyperparameters, dataset, and implement details remained the same.

The confusion matrices were measured and are shown in Figure 6. The testing data set contained facial images of NFW, IFW, and CFW. The method we proposed correctly classified 767 images (with only 10 prediction errors), thus outperforming those without transfer learning or the SR network in every category.

**Figure 7:**
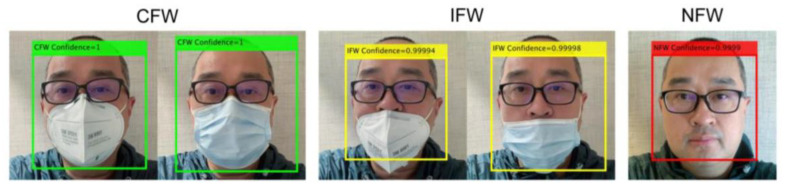
Identification examples. The labels showed the identification results and confidences of SRCNet. CFW = correct facemask-wearing (green), IFW = incorrect facemask-wearing (yellow), NFW = no facemask-wearing (red). The left facial images of CFW and IFW classes are wearing folded facemasks (N95 type), while the right-side facial images are wearing medical surgical masks.

The identification result of different facemasks is illustrated in Table 7. There are generally two types of facemasks: Medical surgical mask and basic cloth face mask, where the facemasks are close to the faces; folded facemasks and the N95 type, where some space is between the face and facemask. An example of these two types of facemasks is demonstrated in Figure 7. The result shows that the SRCNet identifies different types of facemask-wearing conditions with high accuracy.

The performance of SRCNet in different colors of facemasks is also measured, as shown in Table 8. Blue, white, and black are the three most common color for facemasks, while some masks are other colors like green or gray, or patterned. The SRCNet can identify facemask-wearing condition with different facemask colors, which means that the SRCNet is robust.

**Figure 8:**
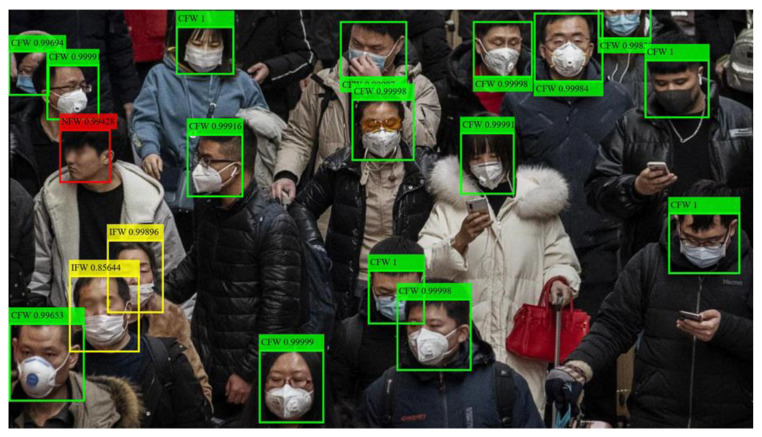

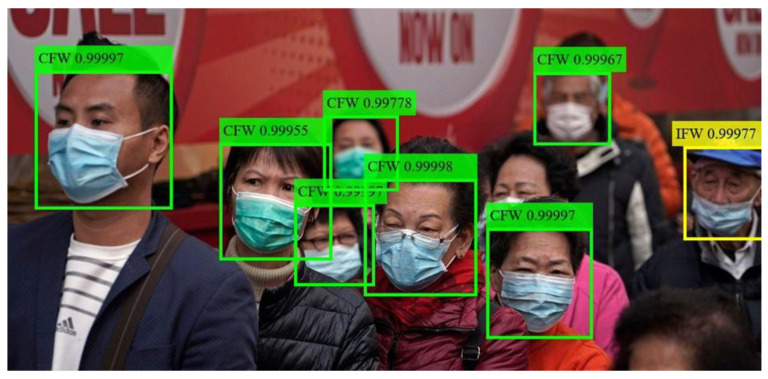
Identification examples in real situations. The labels show the identification results and confidences of SRCNet. CFW = correct facemask-wearing (green), IFW = incorrect facemask-wearing (yellow), NFW = no facemask-wearing (red). The original image was from Medical Masks Dataset (https://www.kaggle.com/vtech6/medical-masks-dataset).

Examples of identification results are shown in Figure 7 and Figure 8. Although the face positions and types of facemasks vary, SRCNet correctly identified all facemask-wearing conditions with high confidence. As analyzed from failed cases, the critical states (wearing facemask between CFW and IFW), image quality, and blocked faces were the three main reasons for identification errors. The ways of wearing facemasks were continuous variables, while the classification results were discrete; hence, critical states were one of the main causes of misidentification. Besides, when the image quality was low (e.g., low-resolution, blocking artifacts, ringing effects, and blurring) or when the faces were partly occluded by objects or other faces, SRCNet had a higher error rate. In addition, SRCNet was likely to make bias errors when the color of a facemask was close to the facial skin color.

The average prediction time of SRCNet was also measured, which was 0.03 s (0.013 s for SR network, and 0.017 s for facemask-wearing condition identification network) for a single face when implemented with MATLAB 2020a, a i7 CPU, and P600 GPU with 4 GB memory. Although the SR process is time-consuming, it improved the SRCNet performance especially in extreme situations.

The processing time for a single image depended on the number of facial images, but, generally, it is much shorter than the sum of faces, as we could use parallel tools to shorten the time. The average processing time for a single image with around six faces was about 0.1 s.

The comparison result with the none-deep learning method is shown in Table 9, including SVM with HOG features and kNN [46,82]. The SRCNet outperformed these methods in both accuracy and kappa. In addition, the SVM and kNN had better performance with images processed by the SR network, which were the same results as the ablation experiment of SRCNet.

## 5. Discussion and Conclusions

### 5.1. Discussion

Our study presented a novel algorithm to identify facemask-wearing condition, which involved four main steps: Image pre-processing, facial detection and cropping, SR, and facemask-wearing condition identification. We proposed SRCNet with a refined SR network to improve its performance on low-quality images. The results indicate that, by using SR before classification, CNNs can achieve higher accuracy. Besides, our experiment proved that deep learning methods can be used to identify facemask-wearing conditions, thus having potential applications in epidemic prevention involving COVID-19.

This study was mainly based on large-scale facial image datasets and the Medical Masks Dataset. For the SR network, we proposed a new network architecture, including improvements in the activation functions and the density of skip connections. These innovations led to considerable performance gains in detail enhancement and image restoration, compared to previous state-of-the-art methods, as evaluated by PSNR and SSIM. The performance of the SR network was also visualized using images with different resolutions, where the proposed SR network restored more details and contributed to the performance of identifying facemask-wearing condition.

For facemask-wearing condition identification, the proposed SRCNet innovatively combined the SR network with a facial identification CNN for performance improvement. Image pre-processing was utilized in SRCNet for better performance, eliminating the irrelevant variables in images such as background, different cameras, exposures, and contrast. In addition, superior detection of the facial area could be achieved with pre-processed images. Transfer learning was also applied during facemask-wearing condition identification network training. Finally, SRCNet achieved a 98.70% accuracy in three-category classifications (NFW, IFW, and CFW) and outperformed traditional end-to-end image classification methods without the SR network by over 1.5% in kappa. An ablation experiment was also conducted to illustrate the effects of transfer learning and the SR network, which were shown to contribute to the final network performances. Identification results in different types of facemasks with different colors were also illustrated to demonstrate the robustness of SRCNet. In addition, none-deep-learning approaches, including SVM and kNN, were also compared and analyzed, where better performances were achieved with the SR network. Our findings indicate that, by using an SR network and a pre-trained general facial recognition model, SRCNet can achieve highly accurate results in identifying facemask-wearing condition.

The identification of facemask-wearing conditions has many similarities to facial recognition. However, the development of a facemask-wearing condition identification network is challenging for several reasons. The limitation in datasets is one main challenge. The facemask-wearing condition datasets are generally small, and their image quality is not high enough, compared to general facial recognition datasets. Furthermore, the various performances of wearing facemasks incorrectly largely increases the difficulty of identification. To overcome these challenges, SRCNet was introduced, which utilizes both an SR network and transfer learning before classification. The SR network solved the low-quality image problem, while transfer learning solved the challenge of using a small dataset with various wearing-facemask-incorrectly examples; with these methods, the performance improved considerably.

To our knowledge, there have not been any studies on facemask-wearing condition identification using deep learning. In our study, facemask-wearing condition was detected with 98.70% accuracy, indicating that SRCNet has great potential to support automatic facemask-wearing condition identification applications. The design of SRCNet also considers network complexity, being based on lightweight and efficient CNNs for real-time facemask-wearing condition identification. The low computing resource requirements of SRCNet mean that it can be applied in public, using internet of things (IoT) technologies, and is meaningful to urge the public to correctly wear facemasks for epidemic prevention.

### 5.2. Conclusions

A new facemask-wearing condition identification method was proposed, which combines an SR network with a classification network (SRCNet) for facial image classification. To identify facemask-wearing condition, the input images were processed with image pre-processing, facial detection and cropping, SR, and facemask-wearing condition identification. Finally, SRCNet achieved a 98.70% accuracy and outperformed traditional end-to-end image classification methods by over 1.5% in kappa. Our findings indicate that the proposed SRCNet can achieve high accuracy in facemask-wearing condition identification, which is meaningful for the prevention of epidemic diseases including COVID-19 in public. There are a few limitations to our study. Firstly, the Medical Masks Dataset we used for facemask-wearing condition identification is relatively small, where it cannot cover all postures or environments. In addition, the dataset does not contain video, where the identification result on a video stream cannot be tested. As for the proposed algorithm, the identification time for a single image is a little long, where an average of 10 images can be identified in a second, which does not meet the basic video frame rate of 24 frames per second (fps). In future studies, a more extensive facemask-wearing data set including images and videos will be collected and labelled with more details, in order to improve the performance of SRCNet. The data set shall contain faces with different postures, environments, and lighting conditions. In addition, SRCNet will be improved, based on either single image or video with IoT technologies, and a more efficient and accurate algorithm will be explored, which can contribute to the practical application of identifying facemask-wearing condition.

## Figures and Tables

**Table 1:** Image resolution statistics.

Resolution N × N	NFW	IFW	CFW	Total
N ≤ 64	307	34	1126	1467
N ≤ 112	199	33	984	1216
N ≤ 150	73	20	355	448
N ≤ 224	77	33	354	464
N > 224	15	14	211	240
Total	671	134	3030	3835

**Table 2:** Average peak signal-to-noise ratio (PSNR) and structural similarity index (SSIM) in different standard deviations σ of gaussian filters.

SSIM						
σ	Proposed SR network	RED	VDSR	SRCNN	Lanczos	Bicubic
5	**0.9423**	0.9390	0.9334	0.9312	0.9198	0.9187
10	**0.9397**	0.9366	0.9313	0.9294	0.9186	0.9176
20	**0.9390**	0.9360	0.9307	0.9290	0.9183	0.9173
30	**0.9389**	0.9358	0.9306	0.9289	0.9183	0.9172
50	**0.9388**	0.9358	0.9305	0.9289	0.9183	0.9172
**PSNR**						
**σ**	**Proposed SR network**	**RED**	**VDSR**	**SRCNN**	**Lanczos**	**Bicubic**
5	**29.3795**	29.0487	29.3189	28.3087	27.6007	27.5618
10	**29.1508**	28.8447	29.1288	28.3934	27.5037	27.4700
20	**29.0887**	28.7877	29.0794	28.1599	27.4790	27.4467
30	**29.0765**	28.7764	29.0692	28.1544	27.4741	27.4421
50	**29.0722**	28.7726	29.0656	28.1525	27.4721	27.4402

**Table 3:** Average PSNR and SSIM in different kernel sizes of gaussian filters.

SSIM						
Kernel Size	Proposed SR network	RED	VDSR	SRCNN	Lanczos	Bicubic
3 × 3	**0.9616**	0.9593	0.9590	0.9489	0.9507	0.9482
5 × 5	**0.9397**	0.9366	0.9313	0.9294	0.9186	0.9176
7 × 7	**0.8820**	0.8808	0.8837	0.8846	0.8895	0.8895
**PSNR**						
**Kernel Size**	**Proposed SR network**	**RED**	**VDSR**	**SRCNN**	**Lanczos**	**Bicubic**
3 × 3	**31.0008**	30.9300	32.3308	29.9178	30.8171	30.5641
5 × 5	**29.1508**	28.8447	29.1288	28.3934	27.5037	27.4700
7 × 7	**25.3330**	25.2739	25.6313	25.5768	25.4915	25.5098

**Table 4:** Average PSNR and SSIM in different down-sampled images.

SSIM						
Down-Sample	Proposed SR Network	RED	VDSR	SRCNN	Lanczos	Bicubic
64 × 64	**0.8984**	0.8974	0.9024	0.8886	0.9110	0.9083
96 × 96	**0.9238**	0.9232	0.9226	0.9237	0.9183	0.9166
112 × 112	**0.9397**	0.9366	0.9313	0.9294	0.9186	0.9176
150 × 150	**0.9311**	0.9293	0.9306	0.9260	0.9172	0.9172
**PSNR**						
**Down-Sample**	**Proposed SR Network**	**RED**	**VDSR**	**SRCNN**	**Lanczos**	**Bicubic**
64 × 64	**25.7461**	25.7343	26.7740	25.9489	27.3168	27.1157
96 × 96	**27.6264**	27.6412	27.8477	27.8157	27.5565	27.4728
112 × 112	**29.1508**	28.8447	29.1288	28.1890	27.5037	27.4700
150 × 150	**28.7863**	28.5277	29.1932	28.3934	27.3703	27.3977

**Table 5:** Performance of facemask-wearing condition identification network.

Method	Depth	Size	Parameters (Millions)	Running Time
**MobileNet-v2** [43]	**53**	**13 MB**	**3.5**	**0.017**
Inception-v3 [80]	48	89 MB	23.9	0.03
DenseNet201 [41]	201	77 MB	20.0	0.12
ResNet50 [38]	50	96 MB	25.6	0.021
DarkNet19 [81]	19	72.5 MB	21.0	0.020
Xception [40]	71	85 MB	22.9	0.043
VGG19 [36]	19	535 MB	138	0.036

Running time: Average time consumption for a single image classification, which is image processing time by the neural network. Image or network loading time is not under consideration. All networks performed the image classification task 20 times with the same bunch of images, and the times taken were averaged.

**Table 6:** Ablation experiment of super-resolution and classification networks (SRCNet).

Method	Accuracy	Facemask-Wearing Accuracy	Personal Protection Accuracy	κ
**1**	**98.70%**	**99.09%**	**98.83%**	**96.22%**
2	98.17%	98.57%	98.44%	94.69%
3	97.91%	98.70%	98.17%	93.90%
4	97.26%	98.57%	97.39%	92.12%

1: Proposed SRCNet. 2: Proposed SRCNet without SR network, which was an end-to-end facemask-wearing condition identification network with transfer learning. 3: Proposed SRCNet without transfer learning. 4: Proposed SRCNet without transfer learning or SR network, which was an end-to-end facemask-wearing condition identification network without transfer learning. All other settings, including hyper parameters, dataset, and implement details remained the same. Accuracy: Accuracy in three categories classification (NFW, IFW, and CFW). Facemask-wearing Accuracy: Accuracy in wearing a facemask (facemask-wearing, NFW). Personal Protection Accuracy: Accuracy in having good personal protection (fail to have personal protection, including NFW and IFW, having personal protection, two categories classification). κ: Kappa in three categories classification.

**Table 7:** Identification result of SRCNet in different types of facemasks.

Facemasks Type	Accuracy	Facemask-Wearing Accuracy	Personal Protection Accuracy	κ
1	98.02%	98.52%	98.27%	95.82%
2	98.99%	99.19%	99.19%	97.72%

**1**: Folded facemasks and N95 type. **2**: Medical surgical masks and basic cloth face masks. Accuracy: Accuracy in three categories classification (NFW, IFW, and CFW). Facemask-wearing Accuracy: Accuracy in wearing a facemask (facemask-wearing, NFW). Personal Protection Accuracy: Accuracy in having good personal protection (fail to have personal protection, including NFW and IFW, having personal protection, two categories classification). κ: Kappa in three categories classification.

**Table 8:** Identification result of SRCNet in different colors of facemasks.

Facemasks Color	Accuracy	Facemask-Wearing Accuracy	Personal Protection Accuracy	κ
Blue	98.84%	99.84%	99.22%	97.72%
White	98.68%	98.94%	98.94%	97.23%
Black	97.81%	98.36%	98.36%	94.82%
Others	98.25%	98.25%	98.69%	96.53%

Facemasks color: The main color of facemasks. Other colors are facemasks in rare mask colors, such as green, pink, gray, or patterned. Accuracy: Accuracy in three categories classification (NFW, IFW, and CFW). Facemask-wearing Accuracy: Accuracy in wearing a facemask (facemask-wearing, NFW). Personal Protection Accuracy: Accuracy in having good personal protection (fail to have personal protection, including NFW and IFW, having personal protection, two categories classification). κ: Kappa in three categories classification.

**Table 9:** Comparison with none-deep learning method.

Method	Accuracy	Facemask-Wearing Accuracy	Personal Protection Accuracy	κ
**SRCNet**	**98.70%**	**99.09%**	**98.83%**	**96.22%**
SVM+SR	83.83%	86.05%	83.83%	45.15%
SVM	83.83%	86.18%	83.83%	42.61%
kNN+SR	88.66%	91.40%	89.44%	64.62%
kNN	85.66%	88.27%	86.57%	54.82%

Accuracy: Accuracy in three categories classification (NFW, IFW, and CFW). Facemask-wearing Accuracy: Accuracy in wearing a facemask (facemask-wearing, NFW). Personal Protection Accuracy: Accuracy in having good personal protection (fail to have personal protection, including NFW and IFW, having personal protection, two categories classification). κ: Kappa in three categories classification.

## Data Availability

The public dataset Medical Masks Dataset is available at https://www.kaggle.com/vtech6/medical-masks-dataset.

## Code Availability

Code used in the present work, including image processing and network structures, is available at https://github.com/BrightQin/SRCNet.
